# 3D-Printed Hydrogel Scaffolds Loaded with Flavanone@ZIF-8 Nanoparticles for Promoting Bacteria-Infected Wound Healing

**DOI:** 10.3390/gels10120835

**Published:** 2024-12-18

**Authors:** Jian Yu, Xin Huang, Fangying Wu, Shasha Feng, Rui Cheng, Jieyan Xu, Tingting Cui, Jun Li

**Affiliations:** 1 State Key Laboratory of Materials-Oriented Chemical Engineering, College of Chemical Engineering, Nanjing Tech University, Nanjing 210009, China; 2 NJTECH University Suzhou Future Membrane Technology Innovation Center, Suzhou 215519, China; 3 Department of General Surgery, The Affiliated Jiangning Hospital of Nanjing Medical University, Nanjing 211199, China

**Keywords:** 3D printing, hydrogel scaffolds, ZIF-8, antibacterial, wound healing

## Abstract

Bacterial-infected skin wounds caused by trauma remain a significant challenge in modern medicine. Clinically, there is a growing demand for wound dressings with exceptional antibacterial activity and robust regenerative properties. To address the need, this study proposes a novel multifunctional dressing designed to combine efficient gas exchange, effective microbial barriers, and precise drug delivery capabilities, thereby promoting cell proliferation and accelerating wound healing. This work reports the development of a 3D-printed hydrogel scaffold incorporating flavanone (FLA)-loaded ZIF-8 nanoparticles (FLA@ZIF-8 NPs) within a composite matrix of κ-carrageenan (KC) and konjac glucomannan (KGM). The scaffold forms a stable dual-network structure through the chelation of KC with potassium ions and intermolecular hydrogen bonding between KC and KGM. This dual-network structure not only enhances the mechanical stability of the scaffold but also improves its adaptability to complex wound environments. In mildly acidic wound conditions, FLA@ZIF-8 NPs release Zn^2+^ and flavanone in a controlled manner, providing sustained antibacterial effects and promoting wound healing. In vivo studies using a rat full-thickness infected wound model demonstrated that the FLA@ZIF-8/KC@KGM hydrogel scaffold significantly accelerated wound healing, showcasing its superior performance in the treatment of infected wounds.

## 1. Introduction

The skin, as the third-largest proliferative organ of the human body, plays an indispensable role in maintaining health through its protective barrier, metabolic functions, and hemostatic mechanisms [1,2,3]. Wound healing is a complex and intricate biological process involving four tightly interconnected and precisely regulated stages: hemostasis, inflammation, proliferation, and tissue remodeling/maturation [4,5,6,7]. Regardless of the condition of connective tissue, whether due to injury, abnormality, or chronic inflammation, it can lead to the formation of chronic wounds, which are often characterized by delayed healing or a complete inability to heal [8,9]. Traditional clinical dressings, such as bandages, gauze, and cotton pads, are widely used in medical practice due to their cost-effectiveness, ease of use, and certain therapeutic effects. However, they are limited by inadequate antibacterial, anti-inflammatory, and antioxidant properties, as well as their inability to maintain an optimal moist wound environment [10,11]. These limitations highlight the urgent need for advanced wound dressings with the following characteristics: (1) high hydrophilicity and permeability to maintain moisture balance in the wound environment; (2) effective gas exchange to promote wound “breathing”; (3) strong microbial barrier properties to prevent bacterial contamination; and (4) integrated drug delivery systems to promote cell proliferation and accelerate wound healing.

In recent years, 3D printing technology has demonstrated immense potential in biomedical applications, particularly in the fabrication of wound dressings [12,13]. Among these, hydrogel-based scaffolds loaded with therapeutic agents have emerged as an ideal choice due to their customizable design and precise controllability [14]. Kappa-carrageenan (KC), a natural polysaccharide, has been widely employed in medical applications for its excellent biocompatibility. It can rapidly form gels under appropriate conditions, providing essential physical support and a moist environment crucial for wound healing. The strong adhesive properties of KC ensure close contact between the dressing and the wound, thereby reducing the risk of infection [15,16]. Additionally, KC serves as an effective drug release carrier, enabling sustained drug delivery to enhance localized therapeutic effects [17]. Similarly, konjac glucomannan (KGM), another natural polysaccharide, exhibits remarkable benefits, including its exceptional water absorption capacity, which helps to maintain a moist wound environment and promotes healing [18,19]. Furthermore, KGM is biodegradable and environmentally friendly. Recent studies have shown that KGM promotes fibroblast proliferation, enhancing tissue repair capabilities, and it possesses antibacterial properties that further reduce infection risks [20]. Thus, the combination of KC and KGM provides not only excellent biocompatibility but also essential physical support and hydration for wounds, fostering cell proliferation and tissue regeneration. The synergistic interactions between the rapid gelation properties of KC and the high water retention of KGM significantly enhance the mechanical stability and biocompatibility of the composite material, offering an optimal solution for wound healing and tissue regeneration, as previously reported for other natural polysaccharides such as chitosan [21].

In addition, flavanone (FLA), a natural flavonoid compound with broad biological activity, has garnered attention for its robust antioxidant, anti-inflammatory, pro-angiogenic, and antibacterial properties. However, the disadvantages of FLV, including its short biological half-life, chemical instability, low bioavailability, and poor water solubility, have limited its application in biomedicine [22]. To address these challenges, researchers have explored the use of functional metal–organic frameworks, such as zeolitic imidazolate framework-8 nanoparticles (ZIF-8 NPs) [23,24], as drug carriers. ZIF-8 NPs possess good biocompatibility and biodegradability, making it applicable for biomedical applications [25,26,27]. Notably, ZIF-8 NPs remain stable under physiological conditions but degrades in acidic environments, enabling controlled drug release [28,29]. Upon degradation, ZIF-8 NPs release Zn^2+^ ions, a potent antibacterial agent that enhances pathogen clearance and effectively prevents bacterial infections during wound healing [30,31]. This combinatory strategy not only addresses the inherent limitations of FLA but also paves the way for new applications of ZIF-8 NPs in the biomedical field, particularly in skin wound dressings.

**Figure 1:**
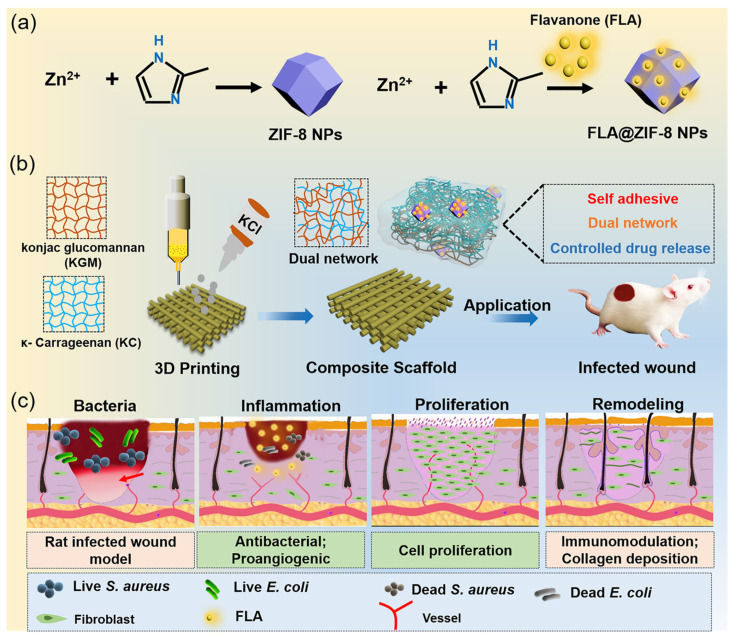
Schematic diagram of 3D-printed hydrogel scaffolds and their application in a rat full-thickness infected wound model. (**a**) Schematic representation of one-pot synthesis of FLA@ZIF-8 NPs. (**b**) Fabrication process of FLA@ZIF-8/KC@KGM hydrogel scaffold using 3D printing technology. (**c**) Effects of FLA@ZIF-8/KC@KGM hydrogel scaffold on the infected wound healing.

In this study, a hydrogel scaffold based on KC and KGM was fabricated using 3D printing technology, with ZIF-8 NPs loaded with FLA incorporated into the hydrogel matrix (denoted as FLA@ZIF-8/KC@KGM). The potential application of this composite material for adjunctive treatment of bacterial-infected wounds was explored (Figure 1). ZIF-8 NPs were synthesized and encapsulated with FLA to form the FLA@ZIF-8 composite, which exhibits high antibacterial properties. Subsequently, KC and KGM were combined with FLA@ZIF-8, and a dual-network structure was formed through the chelation of KC with potassium ions and the hydrogen bonding interactions between KC and KGM. During biodegradation, FLA@ZIF-8 continuously releases Zn^2+^ ions and FLA molecules, particularly in the mildly acidic wound environment, where controlled drug release occurs through self-degradation. In an animal model of full-thickness infected wounds in rats, the FLA@ZIF-8/KC@KGM hydrogel scaffold significantly accelerated wound healing, demonstrating its superior antibacterial efficacy and wound-healing promotion capabilities. Thus, the newly developed composite nanomaterial delivery platform offers a promising and innovative approach for the treatment of infected wounds, addressing the inherent limitations of both FLA and ZIF-8 while also carving a path for the further application of 3D printing technology in the biomedical field.

## 2. Results and Discussion

### 2.1. Synthesis and Characterization of ZIF-8 and FLA@ZIF-8 NPs

**Figure 2:**
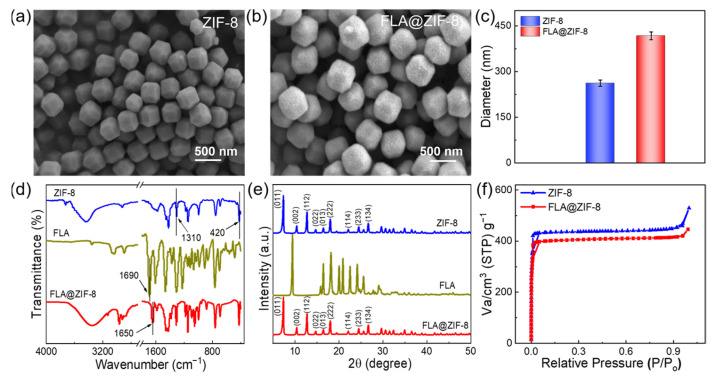
SEM images of (**a**) ZIF-8 and (**b**) FLA@ZIF-8 NPs. (**c**) The average size of ZIF-8 and FLA@ZIF-8 NPs. (**d**) FTIR spectra and (**e**) XRD patterns of ZIF-8 NPs, FLA, and FLA@ZIF-8 NPs. (**f**) Nitrogen adsorption isotherms of ZIF-8 and FLA@ZIF-8 NPs.

In this work, FLA@ZIF-8 NPs serve as high-efficiency antibacterial agents owing to their unique nanostructure and continuous degradation in slightly acidic environments. Figure 2a,b illustrate the scanning electron microscope (SEM) analysis of the synthesized ZIF-8 and FLA@ZIF-8 NPs. The ZIF-8 NPs exhibit nanoscale dimensions, a highly uniform morphology, and a narrow size distribution, adopting an octahedral shape with an average size of approximately 262 ± 10.3 nm (Figure 2c and Figure S1a). The uniform morphology and narrow size distribution are desirable characteristics for drug carriers, facilitating controlled release processes [32]. SEM image of the FLA@ZIF-8 NPs reveals that the incorporation of FLA maintains the structural morphology of the nanoparticles. However, the surface of the FLA@ZIF-8 NPs appears relatively rough, and their average size increases to 417 ± 12.8 nm (Figure 2c and Figure S1b). This observation suggests successful encapsulation of FLA within the ZIF-8 NPs, leading to an increase in particle size. To investigate the structural and chemical properties of the FLA@ZIF-8 NPs in detail, Fourier-transform infrared (FTIR) spectroscopy, X-ray diffraction (XRD), and Brunauer–Emmett–Teller (BET) surface area analyses were conducted. The FTIR spectrum (Figure 2d) identified a C=O stretching vibration of flavanone at 1690 cm^−1^ [33]. In the FTIR spectrum of ZIF-8 NPs, absorption peaks at 1310 cm^−1^ and 421 cm^−1^ were attributed to C-N stretching and Zn-N stretching vibrations, respectively [34]. Notably, in the spectrum of FLA@ZIF-8 NPs, the C=O stretching peak of flavanone shifted from 1710 cm^−1^ to 1650 cm^−1^, providing strong evidence of successful incorporation of FLA into the ZIF-8 NPs. XRD analysis was used to compare the patterns of ZIF-8 NPs, FLA, and FLA@ZIF-8 NPs (Figure 2e). The XRD pattern of ZIF-8 NPs displayed distinct diffraction peaks at 2θ values of 7.32°, 10.36°, 12.68°, 14.72°, 16.4°, 18°, 22.12°, 24.5°, and 26.7°, corresponding to the (011), (002), (112), (022), (013), (222), (114), (233), and (134) planes of ZIF-8 NPs, respectively [35,36]. Notably, the positions of the diffraction peaks of FLA@ZIF-8 NPs are highly consistent with those of ZIF-8 NPs. However, no obvious characteristic peaks of FLA are observed in the XRD pattern of FLA@ZIF-8 NPs, indicating that FLA has been effectively integrated into the crystals of ZIF-8 NPs. Lastly, BET analysis was employed to determine the specific surface areas of ZIF-8 and FLA@ZIF-8 NPs (Figure 2f). ZIF-8 NPs exhibited a high BET surface area of approximately 1309.8 m^2^/g. In comparison, the BET surface area of FLA@ZIF-8 NPs decreased to approximately 1108.2 m^2^/g. This reduction is attributed to the doping of FLA, which likely obstructs the penetration of N2 molecules into the ZIF-8 framework, thereby reducing the surface area of FLA@ZIF-8 NPs [36].

### 2.2. Characterization of the FLA@ZIF-8/KC@KGM Hydrogel Scaffold

**Figure 3:**
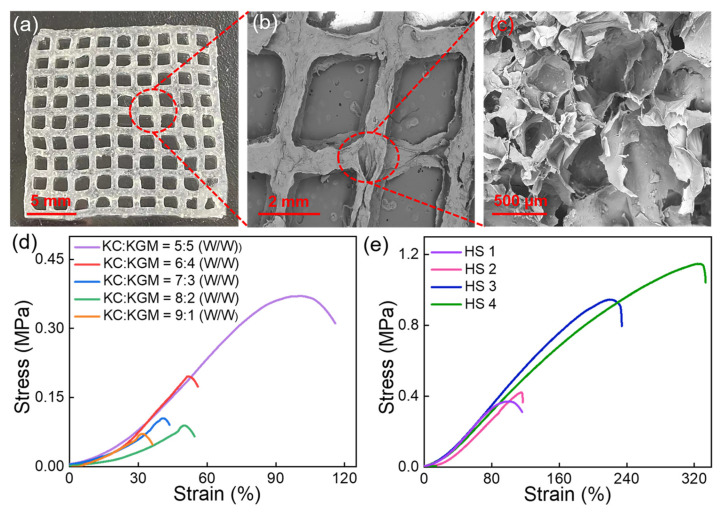
(**a**) Photograph of FLA@ZIF-8/KC@KGM hydrogel scaffold. (**b**,**c**) SEM images of FLA@ZIF-8/KC@KGM hydrogel scaffold. (**d**) Tensile strain curves of hydrogel scaffolds with different KC/KGM mass ratios. (**e**) Tensile strain curves of hydrogel scaffolds with different FLA@ZIF-8 NPs contents.

To advance the innovative application of FLA@ZIF-8 NPs in wound healing, hydrogels were selected as the ideal carrier for loading FLA@ZIF-8 NPs to develop a novel wound dressing. KC and KGM with excellent biocompatibility were selected as hydrogel monomers, and dual-network hydrogels could be formed via electrostatic interactions and ion cross-linking technology [37,38,39]. During the hydrogel preparation process, FLA@ZIF-8 NPs were incorporated to form printing ink for 3D printing FLA@ZIF-8/KC@KGM hydrogel scaffolds. Figure 3a shows a photograph of the FLA@ZIF-8/KC@KGM hydrogel scaffold, exhibiting an orderly grid structure with size of 20 mm × 20 mm × 0.9 mm. SEM images (Figure 3b,c) further confirm the porous structure of the hydrogel scaffold, which favored the controlled release of FLA@ZIF-8 NPs, which was extremely important for promoting wound healing. The water content of the FLA@ZIF-8/KC@KGM hydrogel scaffold was determined to be 98.9%, which benefits from its porous structure. Furthermore, the high water content endows the hydrogel scaffold with superhydrophilic properties, as evidenced by a water contact angle of 22.8° (Figure S2). The mechanical properties of the hydrogel scaffolds were further evaluated, focusing on the impact of different KC-to-KGM mass ratios. Stress–strain tests (Figure 3d) demonstrated that increasing the KGM content enhanced the structural stability and tensile strength of the hydrogel due to the formation of a robust hydrogen-bond network in the hydrogel matrix. The optimal mechanical performance was achieved at a mass ratio of KC to KGM of 5:5, with tensile strength and elongation at break reaching 0.37 MPa and 100.38%, respectively (Figure S3). This ratio was thus selected as the foundational formula for the FLA@ZIF-8/KC@KGM hydrogel scaffold. Subsequently, the effects of FLA@ZIF-8 content on the mechanical properties of the hydrogel scaffolds were investigated. Hydrogel scaffolds containing 0 wt%, 1 wt%, 5 wt%, and 10 wt% FLA@ZIF-8 NPs were prepared and designated as HS 1, HS 2, HS 3, and HS 4, respectively. As shown in Figure 3e, when the content of FLA@ZIF-8 NPs increased from 0 wt% to 10 wt%, the tensile strength increased significantly from 0.37 MPa to 1.15 MPa, and the elongation at break increased significantly from 95% to 320% (Figure S4). The results underscore the dual reinforcing effects of FLA@ZIF-8 NPs in improving both the strength and toughness of the hydrogel scaffolds, highlighting its potential as a multifunctional material for wound healing applications.

### 2.3. Drug Release Performance of FLA@ZIF-8/KC@KGM Hydrogel Scaffold

**Figure 4:**
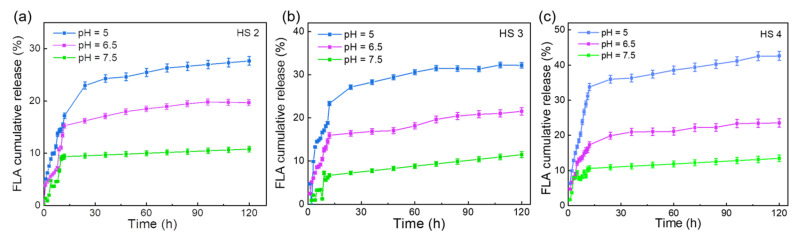
FLA release from (**a**) HS 2, (**b**) HS 3, and (**c**) HS 4 at pH values of 7.5, 6.5, and 5.0. Bars represent standard error, *n* = 3 per group.

To comprehensively evaluate the potential of the FLA@ZIF-8/KC@KGM hydrogel scaffold in drug delivery systems, the release characteristics of FLA from HS 2, HS 3, and HS 4 with different concentrations of FLA@ZIF-8 NPs were investigated under varying pH conditions at room temperature (Figure 4a–c). The results demonstrated that in a simulated physiological environment (pH = 7.5), the HS 2 released approximately 1.33% of the FLA within the first hour. When the pH was lowered to mildly acidic (pH 6.5) and acidic (pH 5.0) conditions, the FLA release increased significantly to 3.76% and 5.08%, respectively. Notably, during the initial 20 h, the release rate of FLA was rapid, nearing its maximum value. After 100 h of monitoring in phosphate-buffered saline (PBS) at pH 7.5, 6.5, and 5.0, the cumulative FLA release reached 10.79%, 19.62%, and 27.67%, respectively (Figure 4a). These results demonstrate the excellent pH-responsive behavior of the FLA@ZIF-8/KC@KGM hydrogel scaffold. The increased release at lower pH conditions highlights the intelligent regulatory capabilities of ZIF-8 NPs as a carrier material, providing targeted drug release in pathological environments. Furthermore, as the content of FLA@ZIF-8 NPs increased, a corresponding improvement in drug sustained release capacity was observed (Figure 4b,c). The release characteristics of Zn^2+^ from HS 2, HS 3, and HS 4 with different concentrations of FLA@ZIF-8 NPs were investigated under varying pH conditions at room temperature (Figure S5a–c). The sustained release behavior of Zn^2+^ in PBS at different pH values is similar to that of FLA. Within the first 15 h, the concentration of Zn^2+^ released increases with the proportion of FLA@ZIF-8 NPs. Furthermore, at the same FLA@ZIF-8 NPs proportion, the release concentration of Zn^2+^ rises as the acidity of the medium increases. This similarity in the sustained release properties of Zn^2+^ and FLA ensures stable synergistic antibacterial and anti-inflammatory effects under specific environmental conditions, thereby promoting the wound healing process. This finding is critical for optimizing drug loading and achieving precise control over release rates. Greater drug release means that the effective drug concentration can be maintained at the wound site for a longer period of time, thus speeding up the wound healing process and improving the treatment effect [40,41]. In summary, with its unique pH responsiveness, FLA@ZIF-8/KC@KGM hydrogel scaffolds bring significant advantages to drug sustained release systems, which not only improve drug targeting and bioavailability but also provide new ideas and technical pathways for realizing personalized medicine and accelerating wound healing.

### 2.4. Antibacterial Properties and Biocompatibility of the FLA@ZIF-8/KC@KGM Hydrogel Scaffold

**Figure 5:**
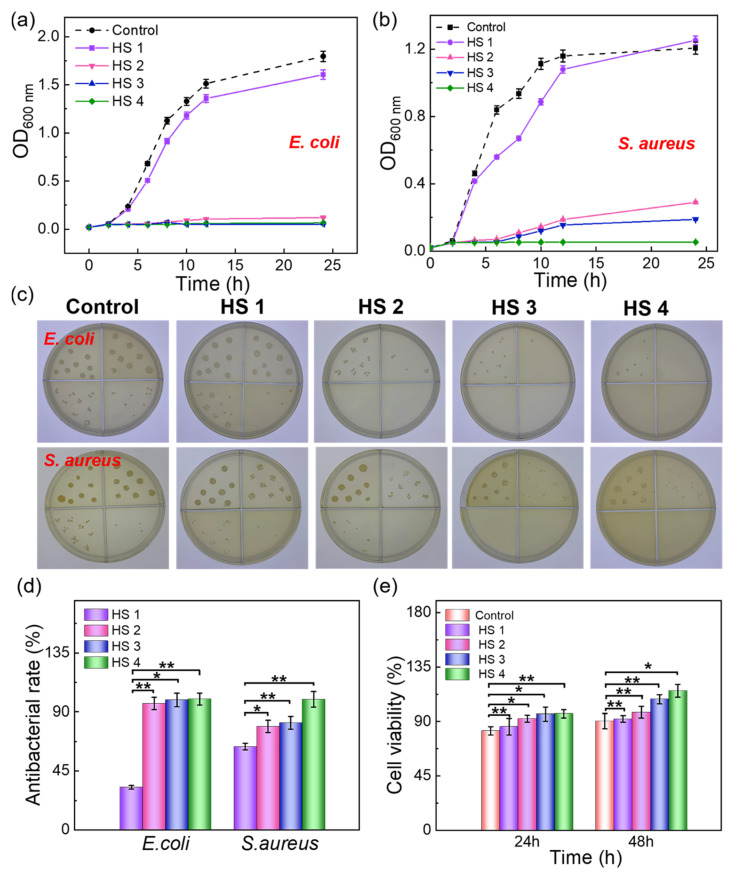
Growth curves of (**a**) *E. coli* and (**b**) *S. aureus* of different groups. (**c**) Photographs and (**d**) antibacterial rates of *E. coli* and *S. aureus* with HS 1, HS 2, HS 3, HS 4 hydrogel scaffolds. (**e**) The cell viability study was performed using L929 fibroblast cells with the MTT assay versus different culture times. Data are shown as mean values ± SD. Bars represent standard error, *n* = 3 per group, * *p* < 0.05, ** *p* < 0.01.

During the wound healing process, the application of dressings with high antibacterial efficacy significantly promotes tissue repair and reduces the risk of infection [42,43]. To explore the antibacterial potential of the FLA@ZIF-8/KC@KGM hydrogel scaffold, control and experimental groups (HS 1, HS 2, HS 3, HS 4) were established to evaluate their antibacterial performance against *Escherichia coli* (*E. coli*) and *Staphylococcus aureus* (*S. aureus*). In the experiment, HS 1, HS 2, HS 3, and HS 4 hydrogel scaffolds were co-incubated with bacterial suspensions containing *E. coli* and *S. aureus*. The optical density at 600 nm (OD_600_) of the bacterial suspensions was monitored over time to quantify the antibacterial activity of each hydrogel scaffold. As shown in Figure 5a,b, the antibacterial performance of the hydrogel scaffolds improved with increasing FLA@ZIF-8 NP contents. To further validate the results, the plate-counting method was employed to evaluate the antibacterial effects of the hydrogel scaffolds. Figure 5c illustrates that the antibacterial activity increased with higher FLA@ZIF-8 NP contents, confirming the superb antibacterial efficacy of FLA@ZIF-8 NPs. The calculated antibacterial efficiency showed that the *E. coli* inhibition rates for HS 1, HS 2, HS 3, and HS 4 were 32.5 ± 1.39%, 96.42 ± 4.69%, 99.12 ± 5.06%, and 99.78 ± 4.45%, respectively, while those for *S. aureus* were 63.63 ± 2.59%, 78.99 ± 4.67%, 81.81 ± 4.85%, and 99.53 ± 5.94%, respectively (Figure 5d). Additionally, biocompatibility is a critical factor in evaluating the safety of medical materials and plays a pivotal role in accelerating wound healing [44,45]. To investigate the biocompatibility of the FLA@ZIF-8/KC@KGM hydrogel scaffold, L929 cells were cultured in media containing extracts from HS 1, HS 2, HS 3, and HS 4 for 24 and 48 h. Live/dead cell staining was obtained via co-culture of L929 fibroblasts with control and experiment groups for 48 h. The fluorescence images (Figure S6) reveal that the L929 fibroblasts in all the groups remained alive with a normal morphology. The MTT assay results showed that after 24 h of co-culture, the cell viability rates for HS 1, HS 2, HS 3, and HS 4 were 85.78%, 92.35%, 96.12%, and 96.35%, respectively. After 48 h, the cell viability increased to 92.12%, 97.65%, 108.35%, and 115.36%, significantly surpassing the control group (Figure 5e). These results demonstrate the excellent biocompatibility of the FLA@ZIF-8/KC@KGM hydrogel scaffolds, highlighting its potential for safe and effective wound healing applications.

### 2.5. Wound Healing Performance of FLA@ZIF-8/KC@KGM Hydrogel Scaffold

**Figure 6:**
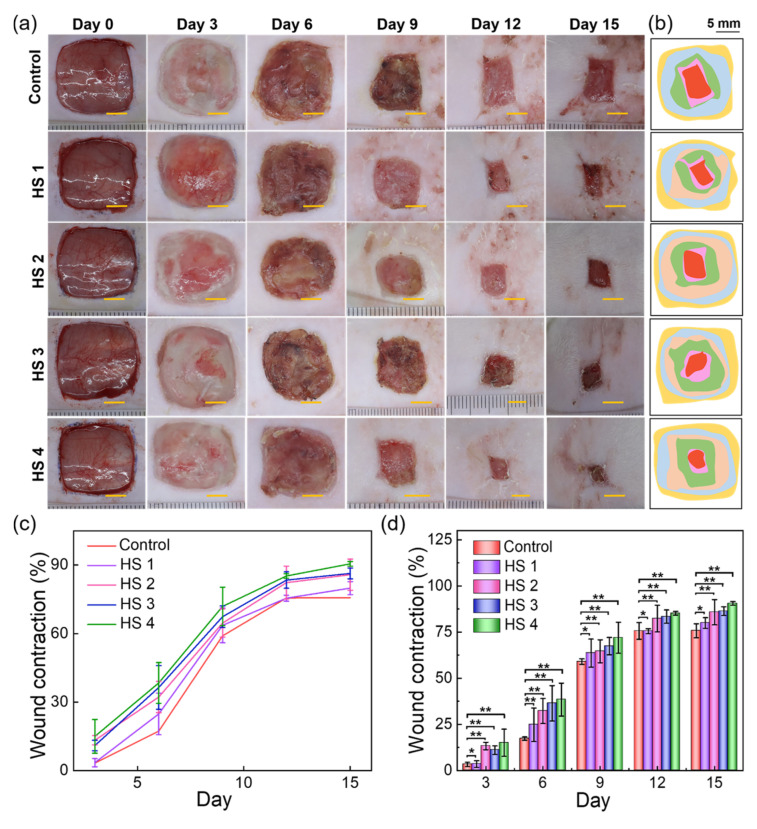
(**a**) Photographs of the wound healing process; (**b**) simulation wound bed closure trace and wound contraction on days 0, 3, 6, 9, 12, and 15 in the control, HS 1, HS 2, HS 3, and HS 4 groups. Scale bar: 5 mm. (**c**,**d**) The wound contraction rates of the control group and the four experimental groups at 0, 3, 6, 9, 12, and 15 days post-treatment. Data are presented as means with standard deviations as error bars, *n* = 5, * *p* < 0.05, ** *p* < 0.01.

The FLA@ZIF-8/KC@KGM hydrogel scaffold demonstrates exceptional potential for treating bacterial-infected skin injuries due to the outstanding antibacterial activity and excellent biocompatibility. To further evaluate the performance for promoting wound healing, a full-thickness skin defect model (1.5 cm × 1.5 cm) was carefully constructed on the backs of rats, and we artificially introduced *E. coli* and *S. aureus* to simulate the infectious wound environment. The wound healing process and treatment effect were systematically tracked to assess the effectiveness of the hydrogel scaffolds. As illustrated in Figure 6a, a control group and four experimental groups were established. The wound healing at 0, 3, 6, 9, 12, and 15 days after implantation of these wound scaffolds was recorded in detail. During the early stages (3 and 6 days), the differences in wound healing areas among the groups were not significant. However, on day 9, all groups showed varying degrees of reduction in wound area. On the 12th day, the HS 4 group showed a particularly prominent healing effect, and the wound recovery was significantly better than that of the control group and the other three experimental groups. At the end of the treatment, on day 15, the wounds treated with the HS 4 hydrogel scaffold were almost completely closed. To further elucidate the healing process, wound bed closure pathways and wound contraction patterns were simulated and visualized (Figure 6b). Wound contraction rates were calculated at each time point based on the wound bed closure trajectory. As shown in Figure 6c,d, after 15 days of treatment, the wound contraction rates for the control, HS 1, HS 2, HS 3, and HS 4 groups reached 75.7 ± 3.8%, 79.89 ± 2.87%, 85.77 ± 6.84%, 86.34 ± 2.38%, and 90.52 ± 1.03%, respectively. These results underscore the exceptional healing capacity of HS 4 during the 15-day treatment period. In summary, the FLA@ZIF-8/KC@KGM hydrogel scaffold significantly enhances wound healing, showcasing its potential as a highly effective treatment for bacterial-infected skin injuries. This finding not only opens new avenues for managing infected wounds but also provides valuable insights for further research in the field of wound healing.

## 3. Conclusions

In this study, an innovative delivery platform that integrates pH-responsive functionalized FLA@ZIF-8 NPs with biocompatible bio-based hydrogel scaffolds was developed to provide highly effective treatment for bacterial infected skin wounds. Using 3D printing technology, the KC@KGM hydrogel scaffold containing FLA@ZIF-8 NPs was carefully fabricated, which ensured the precise and efficient delivery of FLA@ZIF-8 NPs. Notably, these FLA@ZIF-8 NPs exhibit stable decomposition under mildly acidic conditions, enabling the sustained release of FLA and Zn²⁺, thereby demonstrating excellent antibacterial activity. This fabrication strategy not only significantly enhances drug-loading capacity and achieves precise control over drug release but also accelerates the wound healing process. The experimental results show that the FLA@ZIF-8/KC@KGM hydrogel scaffold not only has stable and sustained drug release ability but also has good mechanical strength. In vitro experiments showed that the FLA@ZIF-8/KC@KGM hydrogel scaffold has good biocompatibility, and in vivo experiments verified the significant effects of the hydrogel scaffold in promoting bacterial infection wound healing. The innovative scaffolds developed in this study hold promise as a significant option for skin wound treatment, offering a more efficient, safe, and reliable solution for wound healing in the future.

## 4. Materials and Methods

### 4.1. Materials

K-carrageenan (KC), konjac glucomannan (KGM), potassium chloride (KCl), sodium chloride (NaCl), methanol, 3-(4,5-dimethyl-2-thiazolyl)-2,5-diphenyl tetrazolium bromide (MTT), and phosphate-buffered saline (PBS) were purchased from Shanghai Macklin Biochemical Technology Co., Ltd (Shanghai, China). Zinc acetate dehydrate (ZnAc_2_), 2-methylimidazole, calcein-AM (AM), propidium iodide (PI), and flavanone (FLA) were obtained from Shanghai Aladdin Biochemical Technology Co., Ltd. (Shanghai, China). Ketamine and xylazine were purchased from Beijing Bai Ao Lai Bo Technology Co., Ltd. (Shanghai, China). L929 fibroblasts were procured from the Cell Bank of the Chinese Academy of Sciences (Shanghai, China). The culture medium consisted of 89% Dulbecco’s modified eagle medium (DMEM, Gibco, New York, NY, USA) supplemented with 10% fetal bovine serum (FBS, Gibco, New York, NY, USA) and 1% penicillin–streptomycin solution (Gibco, New York, NY, USA). *Escherichia coli* (*E. coli*, ATCC 25922) and *Staphylococcus aureus* (*S. aureus*, CMCC 26003) were acquired from the Shanghai Preservation Biotechnology Center (Shanghai, China). The deionized water used in the experiments was obtained from Hangzhou Wahaha Group (Hangzhou, China).

### 4.2. Synthesis of ZIF-8 and FLA@ZIF-8 NPs

First, 2.233 g of 2-methylimidazole was dissolved in 10 mL of methanol and stirred at 25 °C for 20 min. Subsequently, 10 mL of a ZnAc_2_ methanol solution (0.6 g/mL) was added to the mixture under vigorous stirring. Within 5 min, the mixture turned milky white. ZIF-8 NPs were collected via centrifugation at 10,000 rpm for 10 min, washed three times with deionized water, and centrifuged under the same conditions to remove residual impurities. The encapsulation of FLA into ZIF-8 NPs was performed using a similar procedure. Specifically, 10 mg of FLA was introduced into the 2-methylimidazole solution during the preparation of ZIF-8 NPs, resulting in the formation of FLA@ZIF-8 NPs.

### 4.3. Construction of FLA@ZIF-8/KC@KGM Hydrogel Scaffold

First, 0.1 g of KC powder was dissolved in 9.9 mL of deionized water and stirred at 80 °C for 1 h to obtain a 1 wt% KC solution. Simultaneously, a 1 wt% KGM solution was prepared by dissolving 0.1 g of KGM in 9.9 mL of deionized water while stirring at 80 °C until completely dissolved. Subsequently, these two solutions were mixed in a 1:1 ratio and stirred at 50 °C to form a homogeneous printing ink. To prepare KC@KGM printing ink containing varying mass concentrations of FLA@ZIF-8 (0, 1, 5, and 10 wt%), the respective amounts of FLA@ZIF-8 NPs were added to the KC@KGM solution. Then, the mixtures were used as printing ink for 3D printing hydrogel scaffolds. After printing, the hydrogel scaffolds were immersed in 50 mL of a 0.5 wt% KCl aqueous solution for 20 min to induce ionic crosslinking between potassium ions and KC. The hydrogel scaffolds were then rinsed three times with deionized water to remove excess ions.

### 4.4. Characterizations

The microstructures of ZIF-8, FLA@ZIF-8 NPs, Fand LA@ZIF-8/KC@KGM hydrogel scaffolds were observed using a field-emission scanning electron microscope (HITACHI S-4800, Tokyo, Japan). The crystal structure of ZIF-8 and FLA@ZIF-8 NPs was analyzed using an X-ray diffractometer (Bruker D8 Advance, Karlsruhe, Germany) with Cu-Kα radiation as the diffraction source. Surface functional groups of FLA, ZIF-8, and FLA@ZIF-8 NPs were characterized using a Fourier-transform infrared (FTIR) spectrometer (Thermo Scientific, Nicolet 6700, Waltham, MA, USA). The pore size distribution of ZIF-8 and FLA@ZIF-8 NPs was measured using a physical adsorption analyzer (Micromeritics, ASAP 2460, Norcross, GA, USA). The mechanical properties of the hydrogel scaffolds were evaluated using a universal testing machine (MTS SANS CMT6203, Shenzhen, China). The image of the testing of the mechanical properties of the hydrogel scaffolds is shown in Figure S7. The water content of the hydrogel scaffold was determined using a moisture rapid measuring instrument (LICHEN LC-DHS-10A, Shanghai, China).

### 4.5. In Vitro Drug Release Assay of FLA@ZIF-8/KC@KGM Hydrogel Scaffolds

The hydrogel scaffolds (HS 2, HS 3, HS 4) were immersed in 30 mL of PBS solutions with different pH values (5.0, 6.5, and 7.5) and agitated continuously at room temperature. At predetermined time intervals, 200 μL of release medium was taken for release profile testing and replaced with an equal volume of fresh medium solution. The absorbance of FLA in the release medium was measured at 427 nm using a TECAN Infinite M200 Pro microplate reader. The concentrations of Zn^2+^ in the release medium were measured using an ion chromatograph (Thermo Scientific, DIONEX ICS-1100, Waltham, MA, USA).

### 4.6. Antibacterial Assay of FLA@ZIF-8/KC@KGM Hydrogel Scaffolds

The antibacterial activity of FLA@ZIF-8/KC@KGM hydrogel scaffolds was evaluated using Gram-positive *S. aureus* (ATCC 25923, Shanghai Preservation Biotechnology Center, Shanghai, China) and Gram-negative *E. coli* (ATCC 25922, Shanghai Preservation Biotechnology Center, Shanghai, China) as model pathogenic bacteria. Quantitative assessment of its antibacterial effect was performed using the plate count method and optical density (OD) measurement at 600 nm.

### 4.7. Cytocompatibility and Cell Viability Assessment of FLA@ZIF-8/KC@KGM Hydrogel Scaffolds

To ensure sterility, hydrogel scaffold samples were subjected to UV irradiation for 30 min. Subsequently, L929 fibroblasts (The Cell Bank of the Chinese Academy of Sciences, Shanghai, China) were seeded into wells containing the hydrogel scaffolds, and DMEM medium supplemented with 10% FBS was used to provide essential nutrients and growth factors. To enhance the reliability of the data, the entire experimental procedure was repeated three times. The cell viability on four different hydrogel scaffolds (HS 1, HS 2, HS 3, HS4) was assessed using live/dead staining. After 48 h of incubation, L929 fibroblasts were stained with AM and PI, following the use of anti-actin antibody (Abcam, Cambridge, UK, 179467) for enhanced visualization. Fluorescence microscopy was employed to observe the live and dead cell populations for each group. Additionally, the viability of L929 fibroblasts was quantified using the MTT assay. Specifically, extracts of hydrogel scaffolds were used to replace the standard DMEM medium for cell culture. After 24 or 48 h of co-incubation, MTT reagent was added to each well, followed by a 2 h incubation period. Absorbance at 490 nm was measured to determine the cell viability.

### 4.8. Animal Modeling and Administration

Sprague–Dawley (SD) rats, aged 8 weeks and weighing 220–260 g, were selected as the experimental subjects. Anesthesia was administered via intraperitoneal injection of a ketamine (50 mg/kg body weight) and xylazine (5 mg/kg body weight) mixture, followed by shaving the back fur to expose the skin. A full-thickness skin wound measuring 1.5 cm × 1.5 cm was created on the back surface of each rat. The rats were then randomly divided into five groups. To simulate an infected wound environment, all wounds were inoculated with *S. aureus* and *E. coli*. After 48 h of infection, different treatments were applied: the control group received no additional treatment, while the other four experimental groups were treated with HS 1, HS 2, HS 3, and HS 4, respectively. Each hydrogel patch matched the wound size (1.5 cm × 1.5 cm) and was applied directly to the wound surface. To ensure experimental independence and accuracy, the rats in each group were housed in separate cages. Wound healing progress was carefully monitored and recorded on days 0, 3, 6, 9, 12, and 15. These observations were used to evaluate the therapeutic effects of the different hydrogel scaffolds.

### 4.9. In Vivo Wound Closure Analysis

In the in vivo wound healing experiment, digital imaging was used to capture the wound areas on five specific days: days 0, 3, 6, 9, 12, and 15. Quantitative analysis of the wound area was performed using digital image analysis software. The percentage of wound closure was calculated based on the following formula:

Wound Closure (%) = (O − A)/O × 100%

where O represents the initial wound area, and A represents the wound area at a given time point. To ensure reliability and reproducibility, three replicates were prepared for each experimental condition.

### 4.10. Ethics Approval

This study involved 25 healthy male Sprague–Dawley rats, each weighing between 180 and 250 g, obtained from Nanjing Medical University, China. This research was conducted in accordance with the guidelines of the Institutional Animal Care and Use Committee (IACUC-20220314-02) at the Jiangsu Center for Safety Evaluation of Drugs, adhering to animal welfare principles as well as all applicable national and local regulations.

### 4.11. Statistical Analysis

The experimental data were analyzed in triplicate and expressed as means ± standard deviations (SDs). Statistical analysis was performed using either an unpaired *t*-test or one-way analysis of variance. A *p*-value of less than 0.05 was considered significant, with * *p* < 0.05, ** *p* < 0.01, and *** *p* < 0.001 indicating different levels of significance.

## Data Availability

The raw data supporting the conclusions of this article will be made available by the authors on request.

## Supplementary Materials

The following supporting information can be downloaded at: https://www.mdpi.com/article/10.3390/gels10120835/s1, Figure S1: Size distribution of (a) ZIF-8 and (b) FLA@ZIF-8 NPs; Figure S2: Water contact angle testing of FLA@ZIF-8/KC@KGM hydrogel scaffold. Figure S3: (a) Tensile strength, and (b) strain of hydrogel scaffolds with different KC:KGM mass ratios; Figure S4: (a) Tensile strength and (b) strain with different hydrogel scaffolds; Figure S5: Zn^2+^ release from (a) HS 2, (b) HS 3, and (c) HS 4 at pH of 7.5, 6.5 and 5.0. Bars represent standard error, *n* = 3 per group. Figure S6: The live/dead staining of control, HS 1, HS 2, HS 3, and HS 4 groups. Scale bar: 400 μm. Figure S7: The image of the testing of the mechanical properties of the hydrogel scaffolds.

## References

[1] Kim H.S., Sun X., Lee J.H., Kim H.W., Fu X., Leong K.W. (2019). Advanced drug delivery systems and artificial skin grafts for skin wound healing. Adv. Drug Deliv. Rev..

[2] Chouhan D., Dey N., Bhardwaj N., Mandal B.B. (2019). Emerging and innovative approaches for wound healing and skin regeneration: Current status and advances. Biomaterials.

[3] Abrigo M., McArthur S.L., Kingshott P. (2014). Electrospun nanofibers as dressings for chronic wound care: Advances, challenges, and future prospects. Macromol. Biosci..

[4] Cui T., Yu J., Wang C.F., Chen S., Li Q., Guo K., Qing R., Wang G., Ren J. (2022). Micro-Gel Ensembles for Accelerated Healing of Chronic Wound via pH Regulation. Adv. Sci..

[5] Cui T., Yu J., Li Q., Wang C.F., Chen S., Li W., Wang G. (2020). Large-Scale Fabrication of Robust Artificial Skins from a Biodegradable Sealant-Loaded Nanofiber Scaffold to Skin Tissue via Microfluidic Blow-Spinning. Adv. Mater..

[6] Yu J., Huang X., Chen X., Hu P., Liu T., Zhang T., Cheng R., Cui T., Li J. (2024). Antibacterial and anti-inflammatory Bi-functional carbon dots hydrogel dressing for robust promotion of wound healing. Carbon.

[7] Sharifi E., Jamaledin R., Familsattarian F., Nejaddehbashi F., Bagheri M., Chehelgerdi M., Zare E.N., Akhavan O. (2023). Bioactive chitosan/poly(ethyleneoxide)/CuFe_2_O_4_ nanofibers for potential wound healing. Environ. Res..

[8] Park H., Patil T.V., Dutta S.D., Lee J., Ganguly K., Randhawa A., Kim H., Lim K.T. (2024). Extracellular Matrix-Bioinspired Anisotropic Topographical Cues of Electrospun Nanofibers: A Strategy of Wound Healing through Macrophage Polarization. Adv. Healthc. Mater..

[9] Xu J., Chang L., Xiong Y., Peng Q. (2024). Chitosan-Based Hydrogels as Antibacterial/Antioxidant/Anti-Inflammation Multifunctional Dressings for Chronic Wound Healing. Adv. Healthc. Mater..

[10] Mistry P., Chhabra R., Muke S., Narvekar A., Sathaye S., Jain R., Dandekar P. (2021). Fabrication and characterization of starch-TPU based nanofibers for wound healing applications. Mater. Sci. Eng. C.

[11] Han Z., Deng L., Chen S., Wang H., Huang Y. (2023). Zn^2+^-Loaded adhesive bacterial cellulose hydrogel with angiogenic and antibacterial abilities for accelerating wound healing. Burns Trauma.

[12] Kim N., Lee H., Han G., Kang M., Park S., Kim D.E., Lee M., Kim M.J., Na Y., Oh S. (2023). 3D-Printed Functional Hydrogel by DNA-Induced Biomineralization for Accelerated Diabetic Wound Healing. Adv. Sci..

[13] Alizadehgiashi M., Nemr C.R., Chekini M., Pinto Ramos D., Mittal N., Ahmed S.U., Khuu N., Kelley S.O., Kumacheva E. (2021). Multifunctional 3D-Printed Wound Dressings. ACS Nano.

[14] Leppiniemi J., Lahtinen P., Paajanen A., Mahlberg R., Metsa-Kortelainen S., Pinomaa T., Pajari H., Vikholm-Lundin I., Pursula P., Hytonen V.P. (2017). 3D-Printable Bioactivated Nanocellulose-Alginate Hydrogels. ACS Appl. Mater. Interfaces.

[15] Feng L., Chen Q., Cheng H., Yu Q., Zhao W., Zhao C. (2022). Dually-Thermoresponsive Hydrogel with Shape Adaptability and Synergetic Bacterial Elimination in the Full Course of Wound Healing. Adv. Healthc. Mater..

[16] Neamtu B., Barbu A., Negrea M.O., Berghea-Neamtu C.S., Popescu D., Zahan M., Miresan V. (2022). Carrageenan-Based Compounds as Wound Healing Materials. Int. J. Mol. Sci..

[17] Chen H., Lan G., Ran L., Xiao Y., Yu K., Lu B., Dai F., Wu D., Lu F. (2018). A novel wound dressing based on a Konjac glucomannan/silver nanoparticle composite sponge effectively kills bacteria and accelerates wound healing. Carbohydr. Polym..

[18] Jiang Y., Huang J., Wu X., Ren Y., Li Z., Ren J. (2020). Controlled release of silver ions from AgNPs using a hydrogel based on konjac glucomannan and chitosan for infected wounds. Int. J. Biol. Macromol..

[19] Yegappan R., Selvaprithiviraj V., Amirthalingam S., Jayakumar R. (2018). Carrageenan based hydrogels for drug delivery, tissue engineering and wound healing. Carbohydr. Polym..

[20] Xu S., Yan S., You J., Wu X. (2024). Antibacterial Micelles-Loaded Carboxymethyl Chitosan/Oxidized Konjac Glucomannan Composite Hydrogels for Enhanced Wound Repairing. ACS Appl. Mater. Interfaces.

[21] Alimirzaei F., Vasheghani-Farahani E., Ghiaseddin A., Soleimani M., Najafi-Gharavi Z. (2017). pH-Sensitive Chitosan Hydrogel with Instant Gelation for Myocardial Regeneration. J. Tissue Sci. Eng..

[22] Kandhare A.D., Ghosh P., Bodhankar S.L. (2014). Naringin, a flavanone glycoside, promotes angiogenesis and inhibits endothelial apoptosis through modulation of inflammatory and growth factor expression in diabetic foot ulcer in rats. Chem. Biol. Interact..

[23] Qin K., Gui Y., Li Y., Li X., Meng F., Han D., Du L., Li S., Wang Y., Zhou H. (2023). Biodegradable Microneedle Array-Mediated Transdermal Delivery of Dimethyloxalylglycine-Functionalized Zeolitic Imidazolate Framework-8 Nanoparticles for Bacteria-Infected Wound Treatment. ACS Appl. Mater. Interfaces.

[24] Rabiee N., Atarod M., Tavakolizadeh M., Asgari S., Rezaei M., Akhavan O., Pourjavadi A., Jouyandeh M., Lima E.C., Mashhadzadeh A.H. (2022). Green metal-organic frameworks (MOFs) for biomedical applications. Microporous Mesoporous Mater..

[25] Zhang S., Ye J., Liu X., Wang G., Qi Y., Wang T., Song Y., Li Y., Ning G. (2022). Dual Stimuli-Responsive smart fibrous membranes for efficient Photothermal/Photodynamic/Chemo-Therapy of Drug-Resistant bacterial infection. Chem. Eng. J..

[26] Nezhad-Mokhtari P., Rahbarghazi R., Hamishehkar H., Asadi P., Milani M. (2024). Innovative Nanocomposite Scaffolds Containing ZIF-8 Nanoparticles for Improving Wound Healing: A Review. J. Polym. Environ..

[27] Yin L., Tang Q., Ke Q., Zhang X., Su J., Zhong H., Fang L. (2023). Sequential Anti-Infection and Proangiogenesis of DMOG@ZIF-8/Gelatin-PCL Electrospinning Dressing for Chronic Wound Healing. ACS Appl. Mater. Interfaces.

[28] Zou Y., Wang P., Zhang A., Qin Z., Li Y., Xianyu Y., Zhang H. (2022). Covalent Organic Framework-Incorporated Nanofibrous Membrane as an Intelligent Platform for Wound Dressing. ACS Appl. Mater. Interfaces.

[29] Reddy Y.N., De A., Paul S., Pujari A.K., Bhaumik J. (2023). In Situ Nanoarchitectonics of a MOF Hydrogel: A Self-Adhesive and pH-Responsive Smart Platform for Phototherapeutic Delivery. Biomacromolecules.

[30] Tang H., Yu Y., Zhan X., Chai Y., Zheng Y., Liu Y., Xia D., Lin H. (2024). Zeolite imidazolate framework-8 in bone regeneration: A systematic review. J. Control. Release.

[31] Yao S., Chi J., Wang Y., Zhao Y., Luo Y., Wang Y. (2021). Zn-MOF Encapsulated Antibacterial and Degradable Microneedles Array for Promoting Wound Healing. Adv. Healthc. Mater..

[32] Zhang W., Zhou Y., Fan Y., Cao R., Xu Y., Weng Z., Ye J., He C., Zhu Y., Wang X. (2022). Metal-Organic-Framework-Based Hydrogen-Release Platform for Multieffective Helicobacter Pylori Targeting Therapy and Intestinal Flora Protective Capabilities. Adv. Mater..

[33] Ibrahim A.-R., Abul-Hajj Y.J. (1990). Microbiological transformation of (±)-flavanone and (±)-isoflavanone. J. Nat. Prod..

[34] Japip S., Erifin S., Chung T.S. (2019). Reduced thermal rearrangement temperature via formation of zeolitic imidazolate framework (ZIF)-8-based nanocomposites for hydrogen purification. Sep. Purif. Technol..

[35] Zhou K., Mousavi B., Luo Z., Phatanasri S., Chaemchuen S., Verpoort F. (2017). Characterization and properties of Zn/Co zeolitic imidazolate frameworks vs. ZIF-8 and ZIF-67. J. Mater. Chem. A.

[36] Barjasteh M., Mohsen Dehnavi S., Ahmadi Seyedkhani S., Yahya Rahnamaee S., Golizadeh M. (2022). Synergistic Wound Healing by Novel Ag@ZIF-8 Nanostructures. Int. J. Pharm..

[37] Brenner T., Tuvikene R., Fang Y., Matsukawa S., Nishinari K. (2015). Rheology of highly elastic iota-carrageenan/kappa-carrageenan/xanthan/konjac glucomannan gels. Food Hydrocolloid..

[38] Hu Y., Tian J., Zou J., Yuan X., Li J., Liang H., Zhan F., Li B. (2019). Partial removal of acetyl groups in konjac glucomannan significantly improved the rheological properties and texture of konjac glucomannan and kappa-carrageenan blends. Int. J. Biol. Macromol..

[39] Penroj P., Mitchell J.R., Hill S.E., Ganjanagunchorn W. (2005). Effect of konjac glucomannan deacetylation on the properties of gels formed from mixtures of kappa carrageenan and konjac glucomannan. Carbohydr. Polym..

[40] Heher P., Muhleder S., Mittermayr R., Redl H., Slezak P. (2018). Fibrin-based delivery strategies for acute and chronic wound healing. Adv. Drug Deliv. Rev..

[41] Li H., Li B., Lv D., Li W., Lu Y., Luo G. (2023). Biomaterials releasing drug responsively to promote wound healing via regulation of pathological microenvironment. Adv. Drug Deliv. Rev..

[42] Xu J., Huang H., Sun C., Yu J., Wang M., Dong T., Wang S., Chen X., Cui T., Li J. (2024). Flexible Accelerated-Wound-Healing Antibacterial Hydrogel-Nanofiber Scaffold for Intelligent Wearable Health Monitoring. ACS Appl. Mater. Interfaces.

[43] Chen X., Huang H., Song X., Dong T., Yu J., Xu J., Cheng R., Cui T., Li J. (2024). Carboxymethyl chitosan-based hydrogel-Janus nanofiber scaffolds with unidirectional storage-drainage of biofluid for accelerating full-thickness wound healing. Carbohydr. Polym..

[44] Mouthuy P.A., Snelling S.J.B., Dakin S.G., Milkovic L., Gasparovic A.C., Carr A.J., Zarkovic N. (2016). Biocompatibility of implantable materials: An oxidative stress viewpoint. Biomaterials.

[45] Xu H., Fang Z., Tian W., Wang Y., Ye Q., Zhang L., Cai J. (2018). Green Fabrication of Amphiphilic Quaternized beta-Chitin Derivatives with Excellent Biocompatibility and Antibacterial Activities for Wound Healing. Adv. Mater..

