# Supplementary material for: 3D-Printed Hydrogel Scaffolds Loaded with Flavanone@ZIF-8 Nanoparticles for Promoting Bacteria-Infected Wound Healing
Source: Gels. 2024 Dec 18;10(12):835. doi: 10.3390/gels10120835 (PMC11675755; doi:10.3390/gels10120835)
Supplement: Supplementary file 1 [file gels-10-00835-s001.zip › gels-3361392-supplementary.pdf]

Supporting Information

3D Printed Hydrogel Scaffolds Loaded with Flavanone@ZIF-8 Nanoparticles for Promoting Bacteria-Infected Wound Healing

Jian Yu[1], Xin Huang[1], Fangying Wu[1], Shasha Feng[1,3], Rui Cheng[1], Jieyan Xu[2]*, Tingting Cui[2,3], Jun Li[2]*

[1]State Key Laboratory of Materials-Oriented Chemical Engineering, College of Chemical Engineering, Nanjing Tech University, Nanjing, 210009, China

[2]Department of General Surgery, The Affiliated Jiangning Hospital of Nanjing Medical University, Nanjing, 211199, China

[3]NJTECH University Suzhou Future Membrane Technology Innovation Center, Suzhou 215519, China

*Corresponding author: R. Cheng (crui0309@njtech.edu.cn); J. Xu (xurn3298@163.com);

Supporting Figures

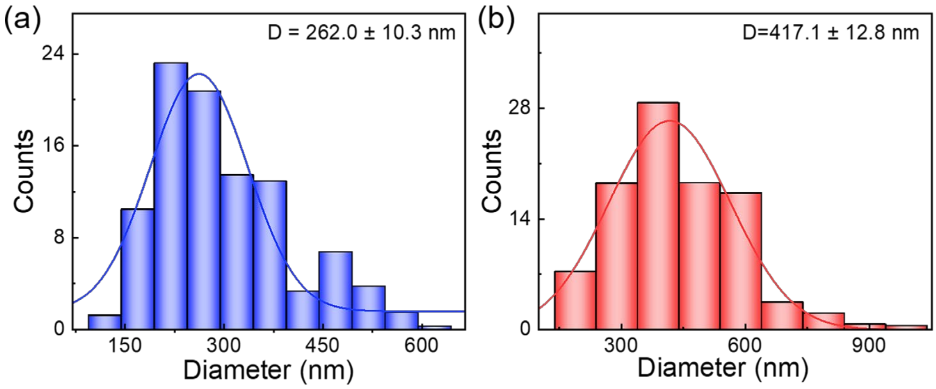

Figure S1. Size distribution of (a) ZIF-8 and (b) FLA@ZIF-8 NPs.

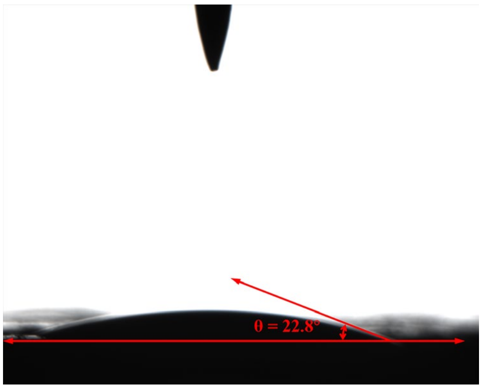

Figure S2. Water contact angle testing of FLA@ZIF-8/KC@KGM hydrogel scaffold.

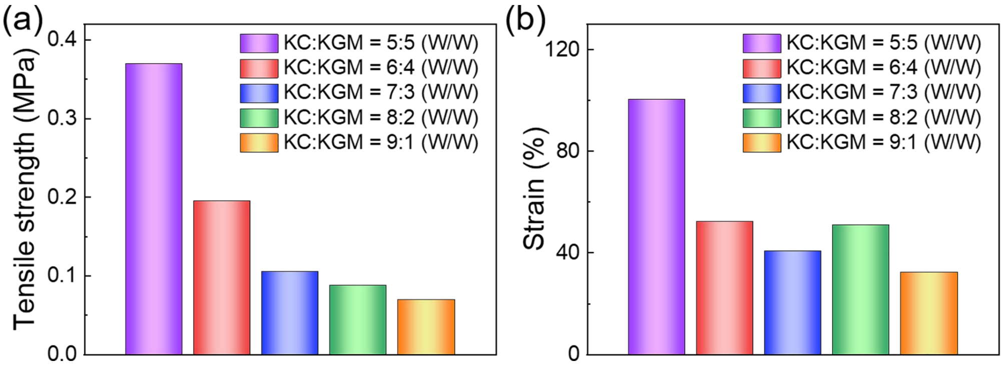

Figure S3. (a) Tensile strength, and (b) strain of hydrogel scaffolds with different KC:KGM mass ratios.

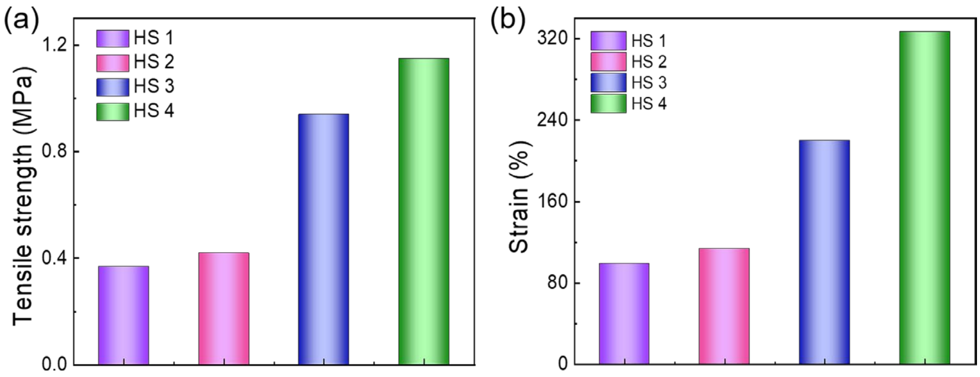

Figure S4. (a) Tensile strength, and (b) strain with different hydrogel scaffolds.

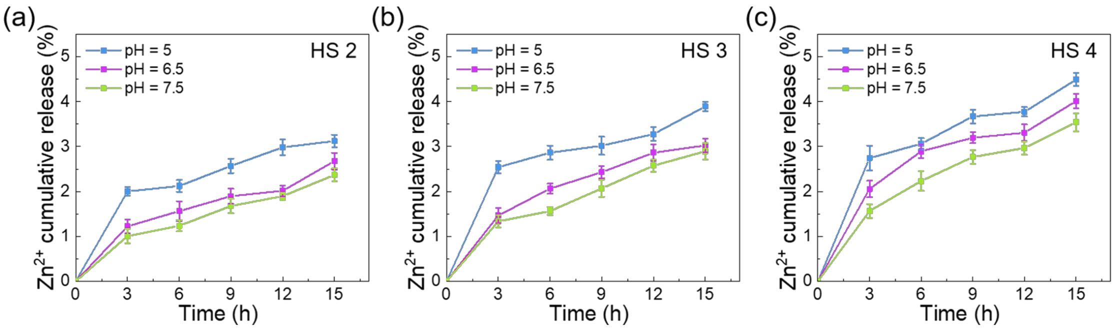

Figure S5. Zn^{2+} release from (a) HS 2, (b) HS 3, and (c) HS 4 at pH of 7.5, 6.5 and 5.0. Bars represent standard error, n = 3 per group.

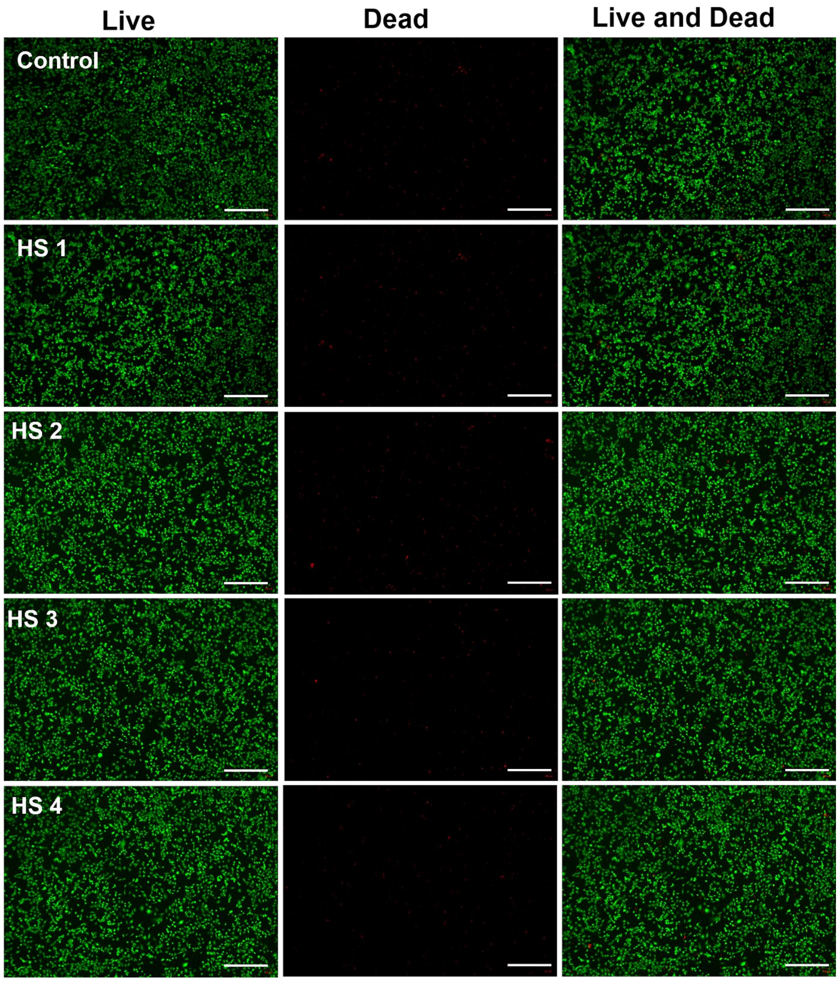

Figure S6. The live/dead staining of control, HS 1, HS 2, HS 3, and HS 4 groups. Scale bar: 400 μm.

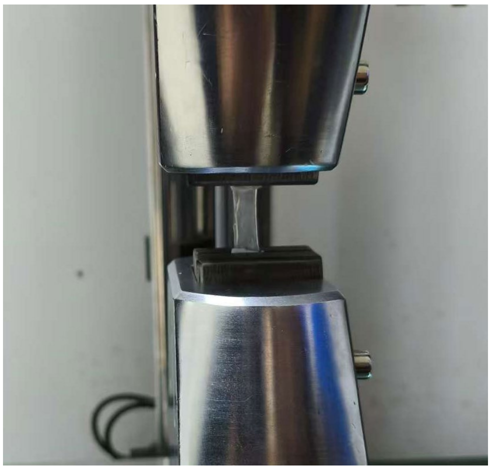

Figure S7. The image of testing the mechanical properties of the hydrogel scaffolds.
